# A Magneto-Hyperelastic Model for Silicone Rubber-Based Isotropic Magnetorheological Elastomer under Quasi-Static Compressive Loading

**DOI:** 10.3390/polym12112435

**Published:** 2020-10-22

**Authors:** Yanliang Qiao, Jiangtao Zhang, Mei Zhang, Lisheng Liu, Pengcheng Zhai

**Affiliations:** Hubei Key Laboratory of Theory and Application of Advanced Materials Mechanics, School of Science, Wuhan University of Technology, Wuhan 430070, China; QiaoYanliang@whut.edu.cn (Y.Q.); zhangmei@whut.edu.cn (M.Z.); liulish@whut.edu.cn (L.L.); pczhai@126.com (P.Z.)

**Keywords:** magnetorheological elastomer, magnetorheological effect, quasi-static compression, magneto-hyperelastic model

## Abstract

A new magneto-hyperelastic model was developed to describe the quasi-static compression behavior of silicone rubber-based isotropic magnetorheological elastomer (MRE) in this work. The magnetization property of MRE was characterized by a vibrating sample magnetometer (VSM), and the quasi-static compression property under different magnetic fields was tested by using a universal testing machine equipped with a magnetic field accessory. Experimental results suggested that the stiffness of the isotropic MRE increased with the magnetic flux density within the tested range. Based on experimental results, a new magneto-hyperelastic model was established by coupling the Ogden hyperelastic model, the magnetization model and the magneto-induced modulus model based on a magnetic dipole theory. The results show that the proposed new model can accurately predict the quasi-static compression property of the isotropic MRE under the tested magnetic flux density and strain ranges using only three model parameters.

## 1. Introduction

Magnetorheological elastomer (MRE) is a kind of smart material composed of micron-sized ferromagnetic particles dispersed in a polymer matrix [1,2,3], which is often referred to as a solid-state analogue of magnetorheological fluid (MRF) [4,5]. It has been shown that the mechanical properties of MRE could be rapidly and reversibly controlled by the external magnetic field due to magnetic dipole-dipole attractions between the magnetized particles [1]. This unique magnetic-field-dependent property makes MRE have numerous potential engineering applications, such as vibration absorber [6], vibration isolator [7], sensing device [8] and other smart MRE devices [9,10].

With the increasing application of MRE in compression mode, both experimental analysis and theoretical modeling are required to predict the response of MRE under quasi-static compressive loading, thus advancing the development of MRE devices. Previous experimental studies have shown that the mechanical responses of MRE are strongly nonlinear functions of magnetic flux density and compressive loading [3,11], which makes it still a substantial challenge to develop constitutive models to describe MRE behaviors. In general, there are two very notable approaches to describe the stress-strain response of MRE under magneto-mechanical coupling conditions, which are microscopic approaches based on the magnetic dipole approximation [1,2,12,13] and macroscopic approaches based on the experimental phenomena [3,14,15,16].

The microscopic dipole approach is generally carried out based on the spatial distribution hypothesis of particles in MRE, and each particle is assumed as a magnetic dipole [1,2]. For example, Jolly et al. [1] developed a simple dipole model in their early work, which was based on the magnetic interactions between two adjacent particles to analyze the MRE properties. Davis [2] proposed another dipole model to predict the magneto-induced modulus by assuming that the particles form an infinite chain in the matrix. Ivaneyko et al. [12] extended the previous approaches and proposed three different lattice models to represent the particle distribution in the MRE. In their works, the magneto-induced deformation and Young’s modulus of MRE were calculated as functions of the external magnetic field strength. Liao et al. [13] proposed a modified magnetic dipole model to investigate the evolution of the normal force of MRE with particle magnetization under the compression state. As found from the above works, magnetic dipole theory microscopically provided some fundamental bases for describing the relationship between the microstructure and mechanical properties of MRE [17]. Unfortunately, the lack of consideration of the reconstruction of particle distribution during MRE deformation makes it difficult to analyze the response of MRE at complex loads, such as large deformations.

For the macroscopic phenomenological approach, previous works mainly focused on developing magneto-mechanical coupling models by fitting experimental results. Varga et al. [3] proposed a phenomenological approach to describe the quasi-static compression property of MRE, in which the magneto-induced elastic modulus was calculated as a function of magnetic flux density. Koo et al. [14] developed a model to capture the compression behaviors of the MRE under cyclic loading by using a multi-layer perceptual artificial neural network. Liao et al. [15] proposed a magneto-mechanical coupling model consisting of hyperelastic, viscoelastic and magnetic components to describe the compression property of MRE under high strain rate. These phenomenological models can be used to predict the MRE behavior under complex loading conditions, even to solve the frequency- and strain-amplitude-dependent problems [16]. However, none of those models had strict microscopic justification or established the explicit functional relationship between the model parameters and the magnetic property [18]. Therefore, it is necessary to develop new phenomenological models to predict the magneto-mechanical coupling behaviors of MRE, which considers such magnetic property and the magnetic field dependence of model parameters.

In this work, a new magneto-hyperelastic model was developed to describe the quasi-static compression property of the isotropic MRE under different magnetic fields. Firstly, a silicone rubber-based isotropic MRE with a particle content of 60 wt.% was prepared, and the magnetization property was tested. The magnetization property of the MRE was described by an empirical model. Then, the quasi-static compression property of the MRE was experimentally tested and modeled using a first-order Ogden model, and a magnetic-field-dependent function of the stiffness parameter in the Ogden model was proposed based on a magnetic dipole model. Combined with the empirical magnetization model, a magneto-hyperelastic compression model of the isotropic MRE as a function of strain and magnetic flux density was finally developed, which was verified by comparing the model predictions with experimental data.

## 2. Materials and Experiments

### 2.1. Materials Preparation

The isotropic MRE used in this paper was prepared by a two-component liquid silicon rubber (type HY-E630, from Shenzhen Hong Ye Jie Technology Co., Ltd., Shenzhen, China) and carbonyl iron particles (CIPs) with an average diameter of 2 μm (type HD-R-1, from Shaanxi Xu Li Heng New Material Co., Ltd., Shaanxi, China). According to the CIP content (60 wt.%) used in MRE and the manufacturer’s recommended weight ratio of the two components (1:1) of silicon rubber, component A, component B and CIPs (A*_weight_*:B*_weight_*:CIPs*_weight_* = 1:1:3) were weighed, respectively. Then, the weighed CIPs were divided into two parts equally and mechanically mixed with component A and component B of silicon rubber for 10 min, respectively. Thus, the CIPs were uniformly dispersed in the two liquid silicon rubber components. After that, the two suspensions were blended together and mechanically mixed for 5 min at the recommended weight ratio of 1:1. The final mixture was poured into the aluminum molds (φ9 mm × 10 mm) and then degassed in a vacuum chamber for 10 min followed by curing at 50 °C for 10 h. After that, the isotropic MRE samples were obtained.

### 2.2. Microstructure, Magnetization Characterization and Compression Experiments

The microstructure of the prepared MRE samples was characterized by scanning electron microscope (SEM) (JSM-7500F from JEOL Ltd., Tokyo, Japan). The sample was first cryogenically fractured in liquid nitrogen and then dried in a vacuum. The fractured surface was coated with carbon powders for SEM observation, and the scanning voltage was set at 5 kV.

The magnetization property of the prepared MRE material in the magnetic field range of 0–1000 mT was tested by using a vibrating sample magnetometer (VSM) (Versalab from Quantum Design Inc., San Diego, CA, USA).

**Figure 1:**
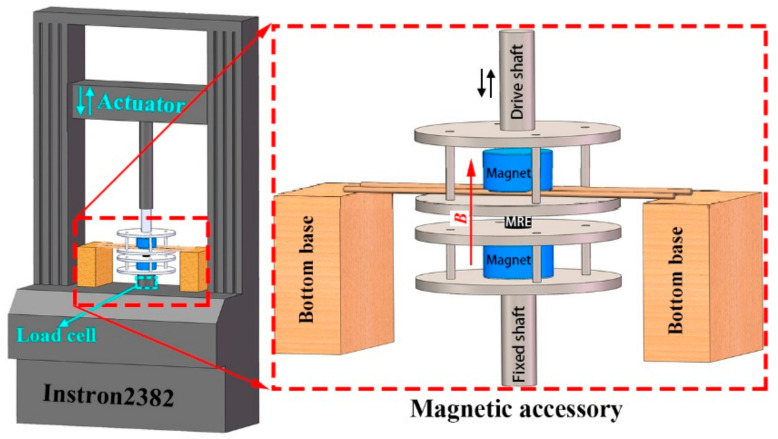
Compression experimental setup.

The compression tests of MRE samples under different magnetic fields were performed using a universal testing machine (Instron2382 from Instron Corporation, Norwood, MA, USA) equipped with a magnetic field accessory, i.e., a test fixture with two fixed permanent magnets, as illustrated in Figure 1. The test fixture was connected to the testing machine via two shafts. Two fixed neodymium permanent magnets (φ40 mm × 20 mm) were used to generate the desired magnetic field with the direction parallel to the axis of the drive shafts. All other parts of the test fixture were made of non-magnetic aluminum alloy with sufficient stiffness. Before each test, the magnetometer probe was placed at the center of the test space, and the magnetic flux density was adjusted by changing the distance between the two magnets. It should be noted that the distance between the magnets remained fixed throughout each test, ensuring a constant magnetic field applied to the samples along the thickness direction.

The compression tests of MRE samples under the magnetic flux densities of 0 mT, 160 mT, 275 mT and 330 mT were tested. The compression speed was set at 0.6 mm/min, and the corresponding initial strain rate was 1 × 10^−3^/s. The samples were compressed to the maximum strain of 30%. To minimize the effect of the friction between the samples and the fixtures, silicone oil was applied to the two ends of the samples during the compression tests. For each magnetic flux density, three measurements were carried out, and the results were averaged based on three measurements.

## 3. Experimental Results and Modeling

### 3.1. Microstructure

**Figure 2:**
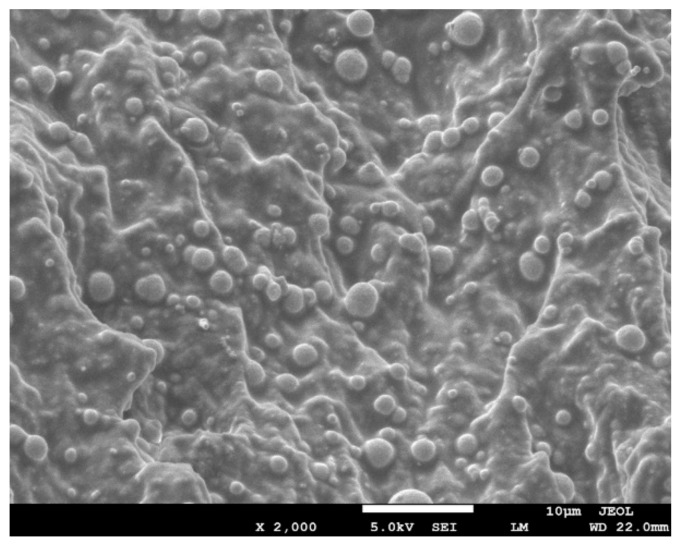
SEM image of the prepared isotropic magnetorheological elastomer (MRE) material.

The SEM image of prepared MRE material is shown in Figure 2. It can be seen that spherical CIPs were uniformly distributed in the matrix, and almost no porosity was observed in the sample. This microstructure suggests that a dense and homogeneous MRE material was obtained in this work.

### 3.2. Magnetization Property and Magnetization Model

**Figure 3:**
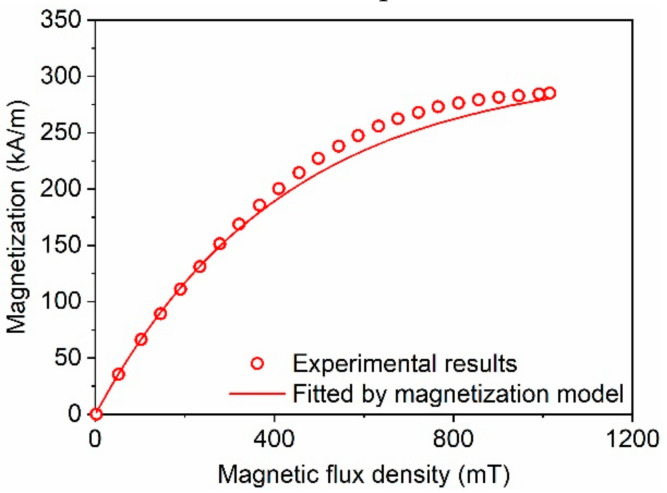
Comparison of the magnetization curves between model calculation and experimental test.

The magnetization curve of the prepared MRE is illustrated in Figure 3. It can be seen that the prepared MRE sample showed the typical magnetization characteristics of soft magnetic material. According to the empirical model proposed by Liu et al. [19], the relationship between the magnetization and the magnetic flux density *B* can be expressed as

(1) $$M_{MRE}\left(B\right)=M_{S}(1-e^{-\chi (B/\mu_{0})})$$

where *M_MRE_* and *M_S_* are the magnetization and saturation magnetization of the MRE material, respectively, *χ* is the adaptive magnetization coefficient, *B* is the magnetic flux density of the external magnetic field and *μ*_0_ = 4π × 10^−7^ H/m is the permeability of vacuum.

Equation (1) is used to fit the magnetization curve of the MRE, and the fitting parameters are as follows: *M_S_* = 307.6 kA/m and *χ* = 3.01 × 10^−6^ m/A. The comparison of the fitted results with the experimental data is shown in Figure 3. The precision of the fitting result was quantitatively evaluated by using the coefficient of determination *R*^2^ [20], which is defined by

(2) $$R^{2}=1-\sum {\left(\Gamma_{\exp}-\Gamma_{\mathrm{mod}}\right)}^{2}/\sum {\left(\Gamma_{\exp}-{\;\bar{\Gamma }}_{\exp}\right)}^{2}$$

where Γ_exp_ and Γ _mod_ are the experimental data and fitting results, respectively. ${\bar{\Gamma }}_{\exp}$ is the average value of the experimental data. When the coefficient of determination *R*^2^ is equal to 1, it indicates that the results calculated by the model are exactly the same as the experimental data. Here, the value of *R*^2^ was 0.9044 in the magnetic field range of 0–1000 mT. However, in the magnetic field range of 0–330 mT, in which the compressive tests of MRE were performed, the value of *R*^2^ was 0.9989.

### 3.3. Compression Experimental Results

**Figure 4:**
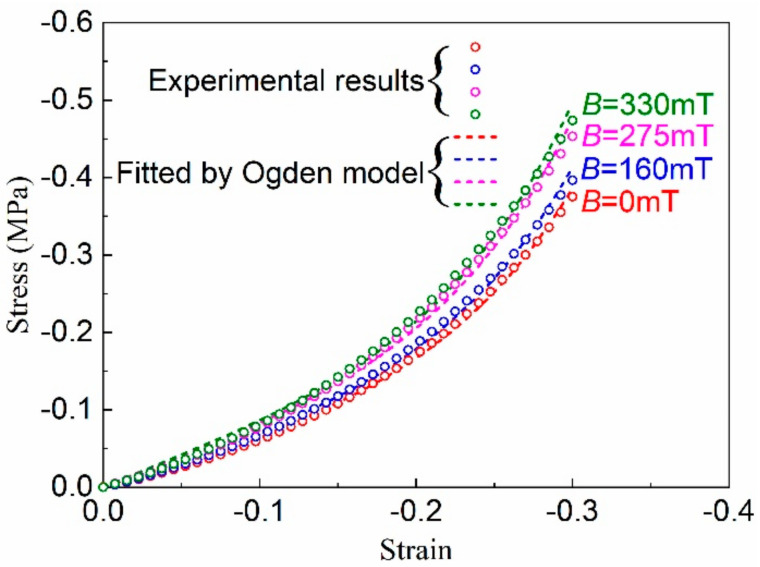
Quasi-static compressive stress-strain curves of the isotropic MRE under different magnetic flux densities.

The quasi-static compressive stress-strain curves of the isotropic MRE under different magnetic flux densities are shown in Figure 4, in which the compressive stress and strain are defined as negative values, and the experimental results are plotted by the color hollow dots. It was found that at a given strain, the compression stress increased with the increasing magnetic flux density. This phenomenon is called the magnetorheological (MR) effect of MRE, which is caused by the magnetic attractions between magnetized particles in MRE under the external magnetic field [1,2]. When applying an external magnetic field, the interparticle magnetic attractions will lead to a three-dimensional network structure of magnetic particles in the MRE, which serves as a reinforcing frame and offers restriction to the mobility of the polymer chains, thus improving the stiffness of MRE under the compressive loading.

As shown in Figure 4, the maximum compressive stress under zero magnetic field was 0.38 MPa at the strain of 0.3, and it was 0.48 MPa under the magnetic flux density of 330 mT. The absolute and percentage increments were 0.10 MPa and 26.3%, respectively, representing the absolute MR effect and the relative MR effect [21]. It could be also seen that the increasing trend of the stress with the strain was basically the same under different magnetic fields, indicating that the strain hardening of MRE under different magnetic fields was controlled by that of the rubber matrix.

### 3.4. Magneto-Hyperelastic Model

In the following sections, the compressive stress-strain curves of MRE material under different magnetic flux densities were first fitted by the Ogden hyperelastic model, and the magnetic field dependence of the stiffness parameter in Ogden model was analyzed. Then, a magnetic field-dependent function of the stiffness parameter was proposed based on a magnetic dipole model. Considering the magnetization model, the magneto-hyperelastic model as a function of strain and magnetic flux density was developed, which was verified by comparing the model predictions with experimental data.

#### 3.4.1. Ogden Hyperelastic Model

According to the strain hardening property of the tested MRE material, the Ogden hyperelastic model is used to fit the compressive stress-strain curves [22,23,24]. The strain-energy function of Ogden model is expressed as the function of the principal stretches λ_1_, λ_2_ and λ_3_, as shown in the following equation [23]:

(3) $$W=\overset{N}{\underset{n=1}{\sum }}\frac{\mu_{n}}{\alpha_{n}}\left(\lambda_{1}^{\alpha_{n}}+\lambda_{2}^{\alpha_{n}}+\lambda_{3}^{\alpha_{n}}-3\right)$$

where *N* is a positive integer, *μ_n_* and *α_n_* are material parameters, and the shear modulus of the material can be calculated by $\mu =\overset{N}{\underset{n=1}{\sum }}\mu_{n}\alpha_{n}$. Under the simple uniaxial loading, the axial stress *σ* is given by the following [25]:

(4) $$\sigma =\overset{N}{\underset{n=1}{\sum }}\mu_{n}\left(\lambda^{\alpha_{n}-1}-\lambda^{-\left(0.5\alpha_{n}+1\right)}\right)$$

where *λ* is the stretch in the loading direction and *λ* = 1 + *ε*. As shown in Equation (4), *α_n_* determines the strain-hardening characteristic of the materials.

Since the stress-strain curves under different magnetic flux densities show the same strain-hardening trend, here we assumed that the strain-hardening parameter *α_n_* is independent of the magnetic field, and it is determined firstly by fitting the Ogden model (Equation (4)) to the stress–strain curve of MRE under the zero magnetic field. It was found that the first-order Ogden (*N* = 1) model was sufficient to obtain an excellent correlation with experimental data. Then, using the determined *α*_1_, the material parameter *μ*_1_ was determined by fitting the first-order Ogden model to the stress–strain curves under different magnetic flux densities. The fitted parameter *α*_1_, the average value of *μ*_1_ and its standard deviation ±Δ*μ*_1_ are listed in Table 1. For comparison, the results fitted by the first-order Ogden (*N* = 1) model are also plotted in Figure 4, as illustrated in Section 3.3. The fitted results agreed well with the experimental results. It can be seen from Table 1 that the stiffness parameter *μ*_1_ increased with the increase of magnetic flux density, reflecting the MR effect of the isotropic MRE material.

#### 3.4.2. Magnetic-Field-Dependent Function of Stiffness Parameter

Since the stiffness parameter *μ*_1_ is the only magnetic-field-dependent parameter in the Ogden model, a magnetic-field-dependent function of *μ*_1_ is needed for the development of a magneto-hyperelastic model for MRE. This is achieved by analyzing the magnetic potential energy of magnetic particles in MRE under the magnetic field. Based on the magnetic dipole model, Ivaneyko et al. [26,27] obtained the magnetic potential energy of magnetic particles in isotropic MRE as a function of the strain ε, which can be expressed by

(5) $$U\left(\varepsilon \right)=\frac{\mu_{0}}{4\pi }{\left(\phi M\right)}^{2}f\left(\varepsilon \right)$$

where *ϕ* and *M* are the volume content and magnetization of CIPs, respectively. Note, that *U*(*ε*) in Equation (5) depends on *ϕ* and *M* through their combination *ϕM*, which is the magnetization of MRE material *M_MRE_*(*B*) = *ϕM* [27]. The magneto-induced modulus Δ*E_m_* for an MRE under the magnetic field then can be obtained as the second derivative of magnetic potential energy to *ε* as follows:

(6) $$\Delta E_{m}=\frac{\mu_{0}}{4\pi }\frac{\partial^{2}f\left(\varepsilon \right)}{\partial \varepsilon^{2}}M_{MRE}^{2}\left(B\right)$$

Equation (6) shows that Δ*E_m_* is quadratically proportional to *M_MRE_*, and the proportionality coefficient is a function of *ε*. Due to the linear relationship between the material stiffness and modulus, the increment of stiffness parameter *μ*_1_ of the Ogden model can also be assumed to be quadratically proportional to the magnetization of MRE and is given by

(7) $$\Delta \mu_{1}\left(B\right)=KM_{MRE}^{2}\left(B\right)$$

where *K* is the material constant and assumed to be independent of *ε* for simplicity. It reflects the dependence of the stiffness parameter on the magnetization of MRE. Thus, the magnetic-field-dependent function of *μ*_1_ can be defined as

(8) $$\mu_{1}\left(B\right)=\mu_{1}^{0}+KM_{MRE}^{2}\left(B\right)$$

where $\mu_{1}^{0}$ is the stiffness parameter of the first-order Ogden model under zero magnetic field.

**Figure 5:**
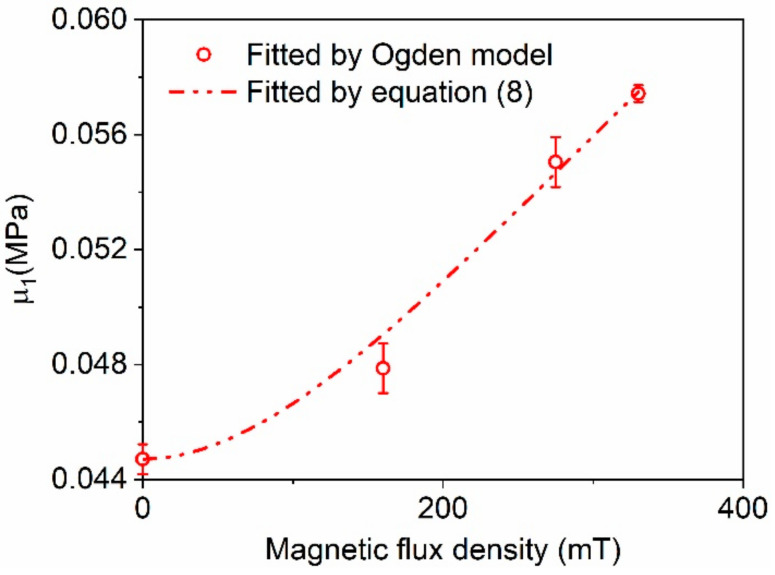
Comparison between the fitting values of stiffness parameter *μ*_1_ and the calculation results using Equation (8).

Combined with Equation (1), Equation (8) was used to fit the data listed in Table 1 by the least mean square (LMS) algorithm, and the obtained value of parameter *K* was 0.45. The comparison between the calculated results by Equation (8) and the fitted parameters listed in Table 1 is shown in Figure 5, in which the standard deviations of *μ*_1_ are also plotted as the error bars. The coefficient of determination *R*^2^ of Equation (8) was 0.9743, indicating that the values of *μ*_1_ could be accurately calculated by Equation (8).

#### 3.4.3. Magneto-Hyperelastic Model and Discussion

Substituting Equation (8) into the first-Ogden model, the magneto-hyperelastic constitutive model is finally obtained as follows:

(9) $$\sigma \left(\varepsilon ,B\right)=\left(\mu_{1}^{0}+KM_{MRE}^{2}\left(B\right)\right)({\left(1+\varepsilon \right)}^{\alpha_{1}-1}-(1+\varepsilon {)}^{-\left(0.5\alpha_{1}+1\right)})$$

**Figure 6:**
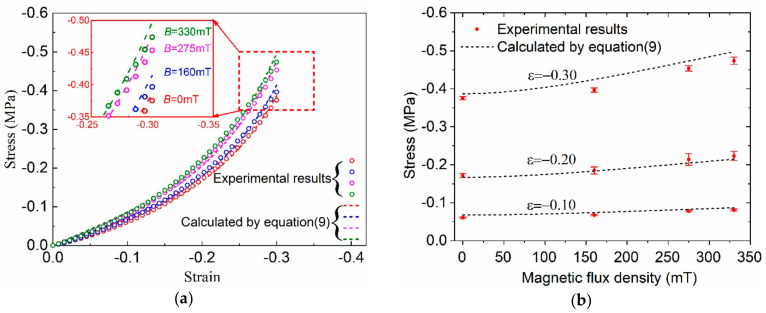
Comparison of the stresses calculated by the magneto-hyperelastic model (Equation (9)) with experimental results: (**a**) stress-strain curves under different magnetic flux densities; (**b**) stress as a function of the magnetic flux density at different strains.

This model has only three parameters, which were determined in the above sections as *K* = 0.45, *α*_1_ = 10.13 and $\mu_{1}^{0}$ = 44,705 Pa. The comparison of the compressive stress-strain curves calculated by Equation (9) with the experimental results is shown in Figure 6a. The coefficients of determination *R*^2^ of the model calculations are 0.9977, 0.9976, 0.9972 and 0.9971 under the magnetic flux densities of 0 mT, 160 mT, 275 mT and 330 mT, respectively. This indicates that the proposed magneto-hyperelastic constitutive model could predict the quasi-static compression property of the prepared isotropic MRE accurately using only three parameters within the tested magnetic flux density and strain ranges. However, it can also be seen from the inserted figure in Figure 6a that the proposed magneto-hyperelastic model overestimated the stresses for the strain close to 0.3 under different magnetic fields. This phenomenon is more clearly shown in Figure 6b, in which the predicted compressive stresses as a function of the magnetic flux density were compared with the experimental results under different given strains. This is because the microstructure changed, such as the debonding of the particle/matrix interfaces, and the reconstruction of particle distribution happened within MRE material under large deformation, causing the decrease of load-bearing ability and the change of the MR effect of the MRE material [17,28]. However, the effects of the microstructure changes were not considered in the present model. In fact, in view of potential practical applications, the strains of MRE material in smart devices usually do not exceed 30% [29].

## 4. Conclusions

In this paper, the magnetization property of a silicone rubber-based isotropic MRE and its compression property under different magnetic fields were experimentally tested at first. It was found that the prepared MRE showed a nonlinear magnetization property and an obvious MR effect. Under the magnetic flux density of 330 mT and the compressive strain of 30%, the absolute and relative MR effects were 0.10 MPa and 26%, respectively. The stress-strain curves under different magnetic flux densities had the same strain-hardening characteristic.

The dependence of the stiffness parameter of the first-order Ogden model on the magnetic flux density was analyzed, and a magnetic-field-dependent function of the stiffness parameter was proposed based on a magnetic dipole model. By combining the first-order Ogden hyperelastic model, the magnetic-field-dependent function of the stiffness parameter and the magnetization model of MRE, a new magneto-hyperelastic model was established. By comparing the model predictions with the experimental results, it was found that the proposed model could accurately predict the compressive stresses of the isotropic MRE within the tested magnetic flux density and strain ranges. The advantage of this magneto-hyperelastic model is that the magnetic-field-dependent compression property of the isotropic MRE can be predicted using only three model parameters, and a constant parameter K that represents the dependence of material stiffness on the magnetization of MRE is defined and can be easily determined. However, the proposed magneto-hyperelastic model overestimates the stresses for the strain over 0.3 due to the lack of consideration of the effects of the microstructure damage and the reconstruction of particle distribution within MRE material under large deformation.

## Figures and Tables

**Table 1:** The fitted model parameters under different magnetic flux densities.

*B* (mT)	*μ*_1_ (Pa)	±Δ*μ*_1_ (Pa)	*α*_1_
0	44,705	524	10.13
160	47,869	864	10.13
275	55,045	869	10.13
330	57,431	291	10.13
